# Unravelling the Collective Calcium Dynamics of Physiologically Aged Astrocytes under a Hypoxic State In Vitro

**DOI:** 10.3390/ijms241512286

**Published:** 2023-07-31

**Authors:** Elena V. Mitroshina, Mikhail I. Krivonosov, Alexander M. Pakhomov, Laysan E. Yarullina, Maria S. Gavrish, Tatiana A. Mishchenko, Roman S. Yarkov, Maria V. Vedunova

**Affiliations:** 1 Institute of Biology and Biomedicine, Lobachevsky State University of Nizhny Novgorod, 23 Gagarin Avenue, 603022 Nizhny Novgorod, Russia; helenmitroshina@gmail.com (E.V.M.); science_pam@mail.ru (A.M.P.); yarullinalia1@mail.ru (L.E.Y.); mary_gavrish@mail.ru (M.S.G.); saharnova87@mail.ru (T.A.M.); roman.sultanov.96@mail.ru (R.S.Y.); 2 Federal Research Center Institute of Applied Physics of the Russian Academy of Sciences (IAP RAS), 603950 Nizhny Novgorod, Russia

**Keywords:** astrocytes, aging, calcium imaging, astrocytes network, hypoxia

## Abstract

Astrocytes serve many functions in the brain related to maintaining nerve tissue homeostasis and regulating neuronal function, including synaptic transmission. It is assumed that astrocytes are crucial players in determining the physiological or pathological outcome of the brain aging process and the development of neurodegenerative diseases. Therefore, studies on the peculiarities of astrocyte physiology and interastrocytic signaling during aging are of utmost importance. Calcium waves are one of the main mechanisms of signal transmission between astrocytes, and in the present study we investigated the features of calcium dynamics in primary cultures of murine cortical astrocytes in physiological aging and hypoxia modeling in vitro. Specifically, we focused on the assessment of calcium network dynamics and the restructuring of the functional network architecture in primary astrocytic cultures. Calcium imaging was performed on days 21 (“young” astrocyte group) and 150 (“old” astrocyte group) of cultures’ development in vitro. While the number of active cells and frequency of calcium events were decreased, we observed a reduced degree of correlation in calcium dynamics between neighboring cells, which was accompanied by a reduced number of functionally connected cells with fewer and slower signaling events. At the same time, an increase in the mRNA expression of anti-apoptotic factor Bcl-2 and connexin 43 was observed in “old” astrocytic cultures, which can be considered as a compensatory response of cells with a decreased level of intercellular communication. A hypoxic episode aggravates the depression of the connectivity of calcium dynamics of “young” astrocytes rather than that of “old” ones.

## 1. Introduction

Brain aging is characterized by a progressive loss of function and deficits in learning and memory. While the cognitive abilities of the brain can be preserved even in old age, aging itself is considered a significant risk factor for the development of neurodegenerative disorders that ultimately lead to senile dementia accompanied by severe cognitive impairment [1]. The cellular mechanisms underlying brain aging are still not fully elucidated.

Astrocytes, a type of glial cell, play a crucial role in maintaining normal brain function. They perform homeostatic functions, including the maintenance of extracellular ion homeostasis, the regulation of energy metabolism of neurons, and neurovascular interaction. Astrocytes are also involved in the regulation of synaptic plasticity and neuronal excitability through the release of neuroactive substances called gliotransmitters [2]. Given their diverse functions, investigating the physiological characteristics of astrocytes is important for gaining insights into the underlying mechanisms of aging and its etiology.

Aging astroglia actively produces mediators typical for inflammation and stress; these alterations have been implicated in the development of neurodegenerative diseases [3]. The role of astrocytes is considered crucial in determining whether the aging process leads to physiological or pathological outcomes. Interestingly, studies over the past decade focusing on neuronal interventions to correct neurodegenerative processes have not yielded significant breakthroughs. As a result, there is growing interest among researchers in targeting astroglia as a potential therapeutic strategy for preventing age-related neurological disorders.

Despite lacking electrical excitability, astrocytes are capable of transmitting intercellular signals to each other. Calcium waves serve as a major mechanism for signal transmission among astrocytes [4]. The generation of calcium signals in astrocytes can be initiated by both neuronal activity and spontaneously. Calcium ions (Ca^2+^) play a vital role in encoding and transmitting information within astrocytes. Calcium waves enable the regulation of the release of gliotransmitters such as glutamate and ATP and affect the expression of various genes. Consequently, these effects can have an impact on neighboring astrocytes, microglial cells, synaptic plasticity, and neuronal excitability [2,5].

A calcium wave refers to a localized increase in cytosolic Ca^2+^ followed by a series of similar waveform events. These calcium waves can occur within a single cell (intracellularly) or propagate to neighboring cells (intercellularly), forming an astrocytic network of activity [6]. Experimental evidence has demonstrated the existence of calcium waves in astrocytes both in vitro and in vivo [7,8,9]. Notably, synchronously propagating calcium waves that pass through several hundred astrocytes have been recorded in vivo in the hippocampus of mice. These waves detected were found to be influenced by the inhibition of purinoreceptors and gap junctions’ intercellular contacts [8]. However, whether calcium waves can be considered as the activity of astrocytic networks rather than individual cells remains a topic of debate. We have previously demonstrated correlated calcium dynamics among astrocytes in vitro, suggesting the existence of a functional network that can change dynamically under different physiological conditions [10,11].

During the 1990s and the early 21st century, disturbances in calcium homeostasis and calcium signaling were proposed as a comprehensive mechanism underlying neuronal aging, which was encapsulated in the “calcium hypothesis of aging” [12,13]. In formulating this hypothesis, neurodegenerative diseases, often viewed as aggravations of the aging process, were conceptualized as “Calciumopathies” [14]. However, a more detailed analysis showed that physiological aging is characterized by more subtle alterations in homeostatic Ca^2+^ mechanisms in neurons making them more susceptible to long-term excitotoxic effects. Conversely, pathological aging associated with neurodegeneration does exhibit aberrant calcium signaling [15,16,17,18]. Recent studies have revealed a distinctive spatial reorganization of Ca^2+^ signals in astrocytes in aged animals [19]. While Ca^2+^ signals in adult mice exhibit a stochastic distribution, in aged animals, these signals become more structured, forming defined Ca^2+^ microdomains with fixed locations. Furthermore, the same study demonstrated that physiological aging is accompanied by morphological atrophy of the peripheral processes of astrocytes in the hippocampus of male C57BL/6 mice [19]. However, the specific features of Ca^2+^ signaling not at the level of a single cell, but within functional astrocytic networks in aged astrocytes remain unknown.

In addition, hypoxia has been identified as a significant contributor to the development of age-related neurodegenerative processes [20] since the brain is highly dependent on a continuous oxygen supply to sustain cellular homeostasis and energy metabolism. Hypoxic damage initiates accelerated cell aging [21,22]. Therefore, the functional response of cells to hypoxic exposure should be studied in the context of age-related changes.

Here, we investigated the characteristics of calcium dynamics and the reorganization of functional network architecture in primary cultures of mouse cortical astrocytes during physiological aging and in the modeled acute normobaric hypoxia in vitro. To simulate the aging process, the primary cortical astrocytes were cultured for a period of 150 days. We also examined changes in the expression levels of pro-inflammatory cytokines, the hypoxia-inducible factor 1 (HIF-1), connexin 43 (i.e., a crucial component of interastrocytic gap junctions), and the anti-apoptotic transcription factor Bcl-2 produced by astrocytes during both aging process and hypoxic damage in vitro.

## 2. Results

### 2.1. Characteristics of Calcium Activity of Physiologically Aged Primary Astrocytic Cultures under Hypoxia

**Figure 1:**
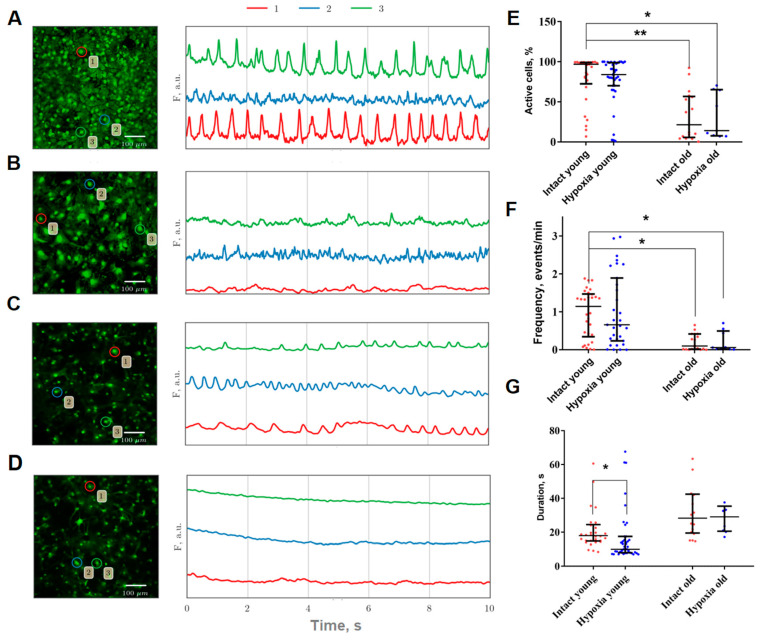
(**A**–**D**): Representative confocal images of primary astrocyte cultures stained with Oregon Green 488 calcium sensor (**left**) and examples of Ca^2+^ profiles of the cells (**right**). The number indicating the cell in the circle corresponds to the color designation of the calcium activity curve. (**A**)—Intact young (21 DIV), (**B**)—Hypoxia young (21 DIV), (**C**)—Intact old (157 DIV), (**D**)—Hypoxia old (157 DIV). Scale bars—100 µm; (**E**–**G**): Main parameters of calcium events occurring in primary cultures of cortical astrocytes. Red or blue dot indicates the mean value of the parameter in a certain culture from the corresponding group. (**E**)—Proportion of cells exhibiting spontaneous calcium activity; (**F**)—Frequency of calcium oscillations; (**G**)—Duration of calcium oscillations. The differences are significant relative to the “Intact young” group: *—*p* ≤ 0.05; **—*p* ≤ 0.01, the Kruskal–Wallis test.

Using original methods developed for imaging data analysis, we studied the features of the functional calcium activity of astrocytes in physiological aging and hypoxia modeling in vitro. Visualization and analysis of the main characteristics of calcium events revealed a statistically significant decrease in the percentage of active cells (i.e., the number of cells in which at least one event was detected during the recording time) and the frequency of occurrence of calcium events by 2.1- and 4.65-times, respectively, compared to young astrocytes (Figure 1A,B).

The hypoxic conditions did not result in a significant change in the proportion of active cells or the frequency of calcium events in both young and old primary cultures of cortical astrocytes. However, the duration of calcium events in young astrocytes was found to decrease during the post-hypoxic period from 18.01 [14.84; 24.52] till 10.06 [8.03; 17.55] (median and interquartile range (IQR)) (Figure 1C).

**Figure 2:**
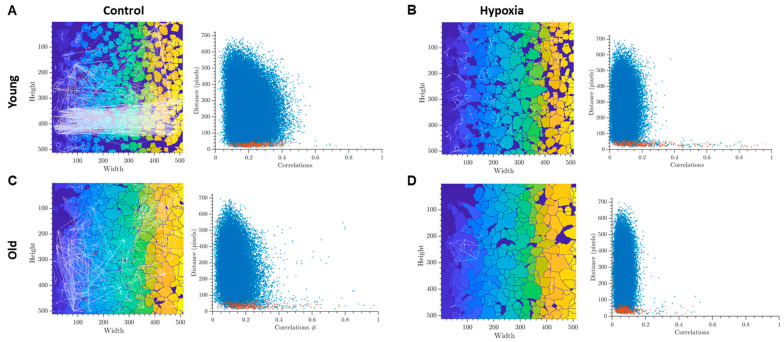
Representative examples of oriented correlation network graphs in primary cultures of cortical astrocytes with a threshold > 0.3 (**left**) and scattergrams (point clouds) (**right**) characterizing the degree of correlation of calcium dynamics between pairs of cells. The colors on the graphs represents a unique identifier of each detected cell region and used for the better visibility of individual cells. Red dots in the scattergrams represent cells that are adjacent to each other, blue dots represent cells that are farther apart: (**A**)—Intact young, (**B**)—Hypoxia young, (**C**)—Intact old, (**D**)—Hypoxia old.

The analysis of correlation-network characteristics of calcium activity provided insights into the collective calcium dynamics in astrocytic networks. By constructing representative correlation network graphs, we visualized the functional relationships between cells and reconstructed the network architecture of astrocytic cultures. We observed that the level of connection of young cultures in the intact group is visually high, while the cultures of this group have the largest number of highly correlated connections (Figure 2A). This indicates a developed dynamic interaction between individual cells and complex network communication. It is known that disturbances in the functioning of astrocytic networks can occur when there is a lack of correlation in the Ca^2+^ fluctuation profile among functionally active cells (Fellin et al., 2006). In the old astrocytes group, distinct changes are observed, particularly a decrease in the correlation of calcium dynamics. This reduction indicates disruptions in calcium intercellular signaling (Figure 2C). In the post-hypoxic period, despite the maintenance of the frequency of calcium event generation and the number of cells exhibiting these events, there is a significant decrease in correlation and dynamic properties of the system in both the “young” and “old” astrocyte groups (Figure 2B,D).

**Figure 3:**
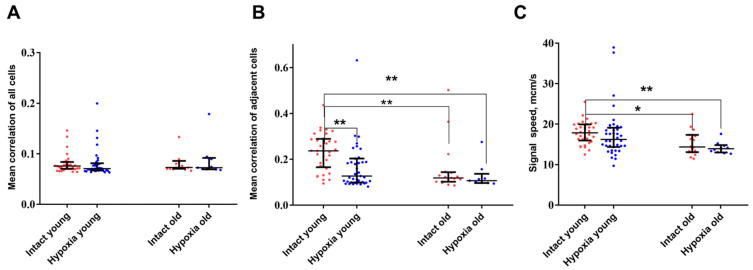
Main network parameters of calcium activity in primary cultures of cortical astrocytes. (**A**)—Average level of correlation of neighboring cells; (**B**)—Average level of correlation of all cells; (**C**)—Signal speed propagation. The computation of speed is conducted only on adjacent cells. The differences are significant relative to the “Intact young” group: *—*p* ≤ 0.05; ** *p* ≤ 0.01, the Kruskal–Wallis test.

The analysis of key connectivity parameters, which describe the network properties of calcium activity, demonstrated that aging leads to a reduction in the correlation of activity among neighboring cells, but not among distant cells (*p* ≤ 0.01). Additionally, a significant decrease in the signal speed propagation was shown (*p* ≤ 0.05) (Figure 3). Furthermore, it is noteworthy that astrocytic networks formed by young and old astrocytes reacted differently to hypoxic injury.

In the post-hypoxic period, young cultures of cortical astrocytes demonstrated a significant decrease in the level of correlation between the activity of neighboring cells. In contrast, the network parameters of calcium activity in the “old” astrocytes group did not exhibit significant changes during the post-hypoxic period. It is plausible to hypothesize that the disruption of the connectivity of calcium dynamics in young cultures in the post-hypoxic period while maintaining the frequency of generation of calcium oscillations and the number of cells in which events are recorded, represents an adaptive response that allows cells to survive the damaging effect.

### 2.2. Features of Signal Transmissions in Physiologically Aged Primary Astrocytic Cultures under Hypoxia

**Figure 4:**
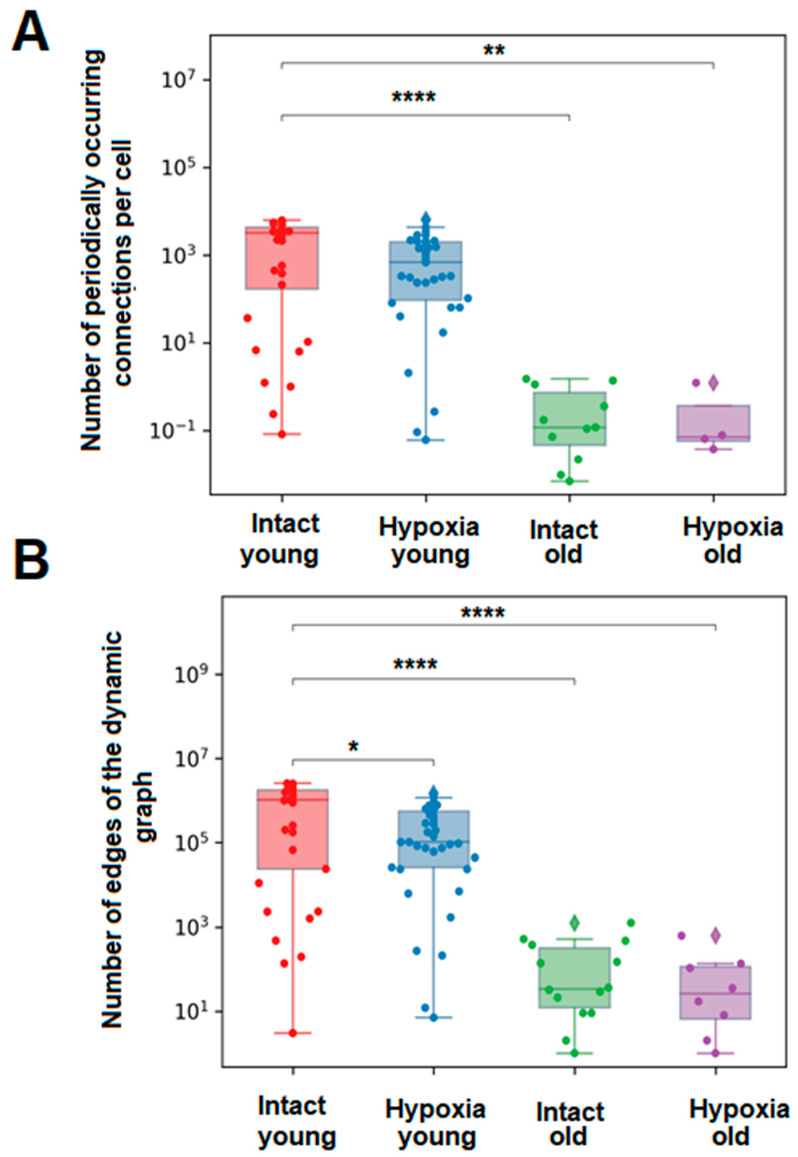
Main parameters of calcium signal transmission in primary cultures of cortical astrocytes. (**A**)—The average number of periodically occurring connections per cell in each group of astrocytes, (**B**)—The average number of edges of the dynamic graph over all frames for each group of astrocytes. The differences are significant relative to the “Intact young” group: *—*p* ≤ 0.05; **—*p* ≤ 0.01; ****—*p* ≤ 0.0001, the Kruskal–Wallis test.

To investigate the effects of aging on the correlation of calcium dynamics within the primary astrocytic culture and the number of signal transmissions between astrocytes, we assessed the number of edges in the dynamic graph, which visually represents the cell-to-cell impulse transmission, and the average number of periodically occurring connections per cell (Figure 4). These parameters provide insights into the significance of individual astrocytes in intercellular communication and the repeatability of signal transmission between specific cell pairs. Supplementary Videos S1 and S2 present examples of dynamic graphs for young and old cultures of astrocytes, respectively. Following hypoxic damage, a significant decrease (*p* ≤ 0.05) in the number of dynamic graph edges was observed in young primary astrocytic cultures, while no significant changes were observed in old cultures (Figure 4).

**Figure 5:**
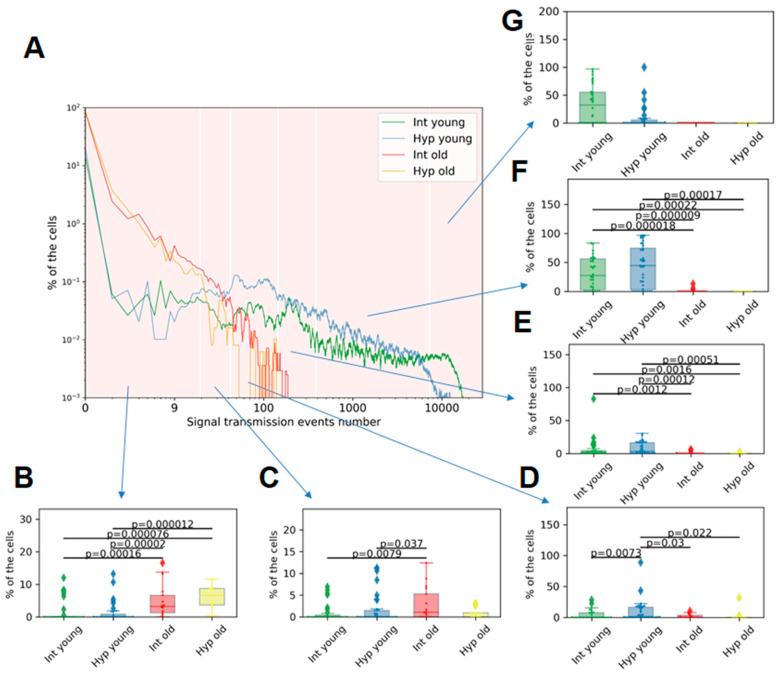
Group histogram of the percentage of cells per number of calcium signaling events in astrocytic cultures: (**A**)—Group histogram—“Intact young” (green), “Hypoxia young” (blue), “Intact old” (red), “Hypoxia old” (orange). (**B**–**G**): Proportion of cells with a number of transmissions in intervals: (**B**)—from 1 to 20; (**C**)—from 21 to 44; (**D**)—from 45 to 150; (**E**)—from 151 to 400; (**F**)—from 401 to 7500; (**G**)—from 7501 to 30,000. Differences are statistically significant at *p* < 0.05, the Kruskal–Wallis test.

The amount of calcium signal transmission in the presented groups of astrocytes was compared using the method of group histograms (Figure 5). For statistical analysis, the horizontal axis of the histogram was divided into six intervals based on the number of transmissions. A statistical analysis of the proportion of cells that fell into each interval was performed.

We observed, for the first time, that young primary cultures of cortical astrocytes exhibit a higher percentage of cells with a larger number of calcium signal transmission events compared to old primary astrocytic cultures. Among the young cultures, only a small proportion of cells (up to 7.88%) had a number of transmissions ranging from 0 to 150 (Supplementary Table S1). In contrast, 1.84 [0.65; 4.41]% of young astrocytes exhibited from 151 to 400 signal transmissions; 27.26 [2.59; 55.66]%—from 401 to 7500 signal transmissions. From 7501 to 30,000 transmissions were registered in 31.96 [0.00; 54.86]% of young astrocytes. The data are presented as the median and IQR. In old astrocytes, a greater prevalence of lower values of signal transmission was observed, with 3.20 [1.23; 6.60]% of cells having 1–44 transmissions and 1.01 [0.00; 5.26]% of cells being active in the range of 21–44 transmissions (Figure 5). Additionally, 45% of cells in old primary astrocytic cultures did not exhibit any recorded events. These findings suggest a significant disruption of calcium communication between cells during physiological aging.

The hypoxic effect on young astrocytes was found to decrease the proportion of cells exhibiting the highest number of signal transmissions. Specifically, 0.00 [0.0000; 4.81]% of cells demonstrate signaling in the range of 7501–30,000 transmissions versus 31.96 [0.00; 54.86]% in the intact group. In contrast, the largest proportion of cells in the “Hypoxia young” group showed signal transmissions in the range of 401–7500 (44.24 [2.65; 74.48]%). No significant differences in the number of signal transmissions were observed in physiologically aged astrocytes during the post-hypoxic period.

### 2.3. Analysis of Hubness and Authority of Physiologically Aged Astrocytes in the Transmission of Calcium Signal

**Figure 6:**
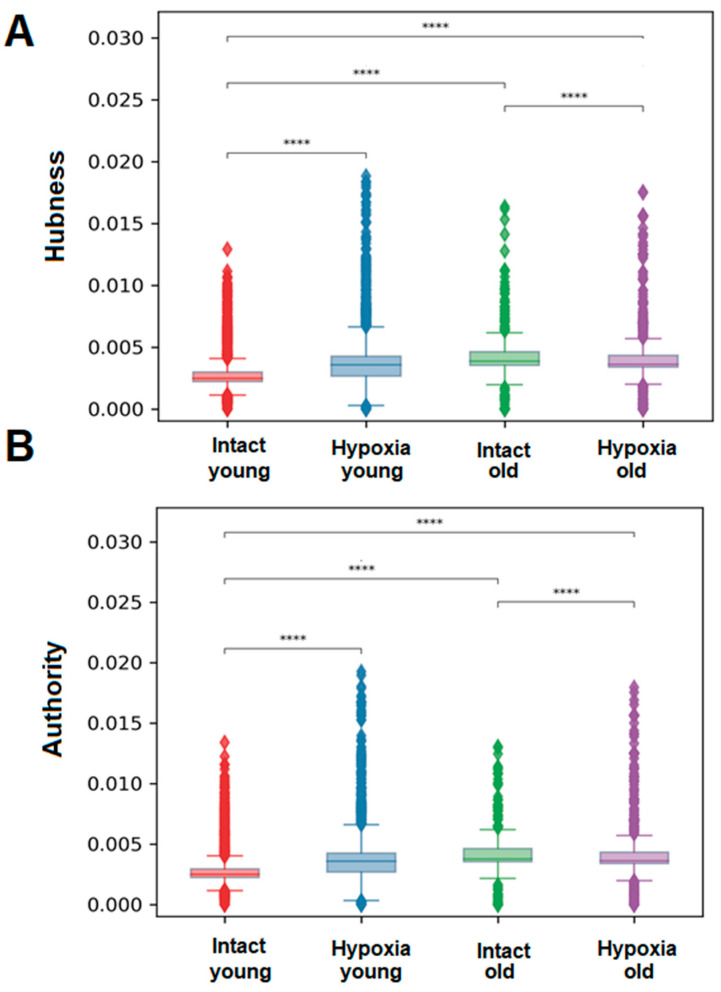
Significance of a cell in signal transmission-the number of transmitting signals and receiving signals by each cell: (**A**)—Mean mediation score of one cell for each astrocytic culture: mediation score can be gained either by transmitting signals to multiple cells or by transmitting signals to one significant cell; (**B**)—Mean value of the significance rating of one cell for each astrocytic culture: the significance rating can be obtained either by receiving signals from multiple cells or by receiving signals from one hub with a high rating, ****—differences are significant compared to the “Intact young” group, *p* ≤ 0.0001, the Kruskal–Wallis test.

It was shown that during physiological aging of primary cultures of cortical astrocytes, both Hubness (significance) and Authority (mediation) metrics were increased. However, in hypoxic damage in the young group of cultures, a significant increase in the values of both metrics is observed, whereas in the old group of cultures, the average values were increased (Figure 6). The metrics of significance and mediation are calculated by assigning scores to each cell in the culture, with the sum of scores equaling one. When there are fewer active cells for any reason, the average significance (Hubness) and culture mediation (Authority) scores increase due to the reduced number of cells participating in the ranking. Consequently, the increase in the values of the metrics of significance and mediation in astrocytes during hypoxic damage and physiological aging is associated with a decrease in the number of signal transmissions and the preservation of active periodic signaling only in a small number of cells in the culture.

### 2.4. Assessment of Secretory Activity of Physiologically Aged Astrocytes

**Figure 7:**
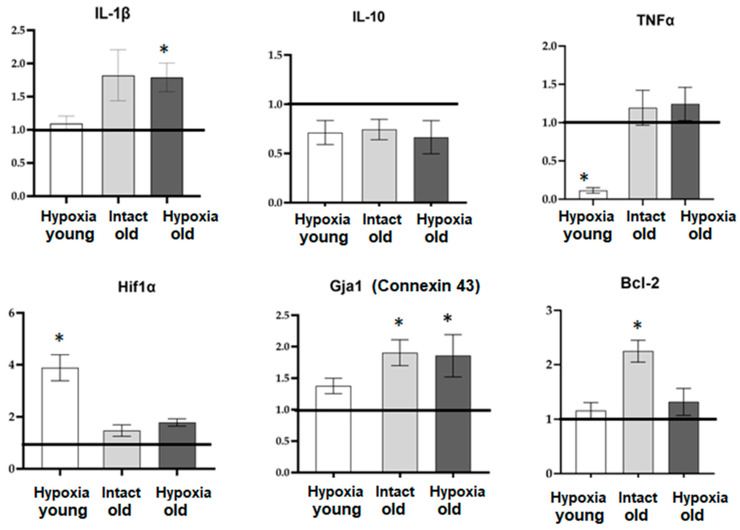
The level of mRNA expression of genes of interest in primary cultures of cortical astrocytes during physiological aging and hypoxia modeling. Data are normalized relative to intact cultures of young astrocytes, and the line at 1.0 reflects data from “Intact young” cells. *—statistically significant difference between the experimental groups, *p* ≤ 0.05, ANOVA one-way analysis of variance and Tukey’s multiple post hoc test.

The changes in the functional activity of astrocytes during physiological aging and hypoxia modeling are likely associated with the transition of astrocytes into a reactive state and alterations in the synthesis of various biologically active substances, particularly pro-inflammatory cytokines and the hypoxia-inducible factor HIF-1, as well as changes in the number of intercellular contacts. In order to assess these changes, we examined the mRNA expression levels of several key regulatory molecules in primary cultures of cortical astrocytes cells during physiological aging and hypoxia modeling using RT-qPCR. The molecules evaluated included the pro-inflammatory cytokines IL-1β and TNF, the anti-inflammatory cytokine IL-10, the hypoxia-induced factor Hif1α, the anti-apoptotic protein Bcl-2, and connexin 43 (*Gja1*), which is one of the important astrocytic connexins. The results of the mRNA expression analysis are presented in Figure 7.

Our findings reveal that aging is associated with an upregulation of mRNA expression of the anti-apoptotic factor Bcl-2 and connexin 43, indicating a compensatory response in cells with reduced intercellular communication. Interestingly, only young astrocytes exhibited an increase in mRNA HIF-1 expression in response to hypoxic damage. This suggests that old astrocytes exhibit profound metabolic disturbances and impaired adaptive capacity. Furthermore, the response of aging astrocytes to hypoxia in terms of pro- and anti-inflammatory factor synthesis also shows alterations. In the post-hypoxic period, young cultures of cortical astrocytes displayed a decrease in the expression of the pro-inflammatory factor TNF, while old astrocytic cultures exhibited an increase in the expression of the pro-inflammatory cytokine IL-1β.

## 3. Discussion

Astrocytic senescence remains a relatively understudied and poorly characterized phenomenon. The morphology and function of aged astrocytes have not been extensively studied. It is generally accepted that the overall number of astrocytes in the central nervous system (CNS) of rodents, primates, and humans does not undergo significant changes with age [23,24,25]. However, atrophy and loss of astrocyte function occur during the aging process [26]. Early studies often reported an increase in the expression of glial fibrillar acidic protein (GFAP), the main structural protein of the astrocyte cytoskeleton, in the CNS as a sign of reactive gliosis in aging rodents [27,28,29] and humans [30]. However, these findings have been challenged, as subsequent studies have reported conflicting results regarding the number of GFAP-positive astrocytes. Several studies have demonstrated an increase in the number of GFAP-positive astrocytes [31,32,33], while others have observed a decrease [34,35] or no change [25]. Additionally, both hypertrophy and atrophy of GFAP-positive astrocytes have been observed with regional specificity [2,31,36,37].

Our understanding of changes in astrocyte physiology during aging is also limited. The primary biophysical properties of astrocyte plasma membranes show minimal alterations, as the resting membrane potential and input membrane resistance do not differ significantly between young and old rodents [38,39]. Astrocytes in aged animals exhibit membrane currents and Ca^2+^ responses to major neurotransmitters, indicating the expression of ionotropic glutamate and P2X receptors, as well as metabotropic glutamate, norepinephrine, cannabinoid, and P2Y receptors [38,40,41].

Our findings align with the existing data, demonstrating that calcium events persist in aged astrocytes. The main focus of our study was to reconstruct the architecture of functional relationships within astrocytic cultures based on calcium imaging data. We have successfully demonstrated a significant decrease in the functional activity and connectivity of aging astrocytes and disrupted intercellular Ca^2+^ signaling in vitro.

Previous studies have provided evidence of age-related changes in the functional expression of key astrocytic receptors. Specifically, it has been observed that the density of ionotropic glutamate and purinergic receptors increases in mice aged from 1 month to 3–6 months but then rapidly declines [38]. Additionally, the amplitude of Ca^2+^ signals elicited by neurotransmitters decreases with age [42]. This reduction in Ca^2+^ signaling is implicated in the age-related decrease in astroglial ATP secretion and the impaired astroglial regulation of metaplasticity [41,43]. This phenomenon may underlie the disruption of intercellular transmission of Ca^2+^ signals and the perturbation of calcium dynamics correlation in glial cells, as we observed here in astrocytes cultured for 157 days in vitro. Our findings indicate that physiological aging exerts a significant inhibitory effect on the calcium activity of primary cultures of cortical astrocytes. We observed a decrease in correlation levels and disturbances in intercellular Ca^2+^ signaling. Specifically, in physiological aging, there is a substantial decrease in the number of signal transmissions by astrocytes, with a reduction of two orders of magnitude (from 10,000 to 20 transmissions), clearly indicating disorganization in the communication between astroglial cells.

Hypoxia is recognized as a significant contributor to the pathogenesis of various neurological disorders, including Alzheimer’s disease, Parkinson’s disease, and other age-related neurodegenerative states [44]. The risk of experiencing both acute and chronic intermittent hypoxia increases with age. Accumulating evidence of recent years supports the notion that a hypoxic or ischemic episode in the brain can serve as a crucial trigger for the onset and progression of dementia and/or Alzheimer’s disease [45,46,47]. Consequently, it is of utmost importance to study the age-related characteristics of neuronal responses to the detrimental effects of hypoxia.

Our findings reveal that hypoxic injury causes significant alterations in the network properties of young primary cortical astrocytes. These changes are characterized by a significant decrease in various connectivity parameters, including the number of signal transmissions, while the number of cells generating calcium events and its frequency remain relatively stable. On the other hand, the impact of hypoxia on calcium signaling in old astrocytes is comparatively less pronounced and fails to reach statistical significance. Nevertheless, both young and aging astrocytes experience a reduction in the correlation and dynamic properties of the system.

Aging leads to increased production of reactive oxygen species and the development of oxidative stress, which can trigger the secretion of a group of factors known as senescence-associated secretory phenotype (SASP) [48,49]. Senescent astrocytes, in particular, release SASP mediators such as IFNγ, CXCL10, IL-6, and TGFβ, which have the potential to induce inflammation [50,51]. Notably, IFNγ, a potent regulatory cytokine, has been implicated in activating microglia and promoting brain inflammation in Alzheimer’s disease [52]. Another typical SASP factor, IL-6, is commonly upregulated in the aging brain and patients with Alzheimer’s disease, and its overexpression has been demonstrated to induce neurodegeneration in vitro [50]. Several SASP factors, including IL-6, IL-1β, TNF-α, MMP-1, MMP-3, and MMP-10, have also been found to be elevated in the cerebrospinal fluid and serum of patients with Alzheimer’s disease [53,54]. These findings suggest that senescent astrocytes contribute to a pro-inflammatory state through the production of SASP factors, and SASP-mediated microglial activation and inflammation may contribute to the pathogenesis of neurodegenerative processes, particularly in Alzheimer’s disease.

However, SASP may differ in senescent astrocytes in normal aging and during the development of age-associated neurodegeneration [35,55]. An important discovery of recent years is that reactive astrocytes are a heterogeneous group of cells, changes in which depend on the specific state and events occurring in CNS pathologies. To differentiate between various cell phenotypes, reactive astrocytes are commonly classified into A1 and A2 types, with A1 astrocytes considered potentially destructive and neurotoxic, while neuroprotective A2 astrocytes are believed to contribute to neuronal survival and maintenance [2,56].

The increase in the mRNA expression of anti-apoptotic factor Bcl-2 and connexin 43, as observed in our study, suggests that during physiological aging, astrocytes may activate the A2 phenotype. It is worth noting that hypoxic exposure induces opposite changes in the secretion profile of biologically active compounds by young and old astrocytes. In young cultures, the decrease in expression of the pro-inflammatory factor TNF during the post-hypoxic period indicates the neuroprotective A2 phenotype. Conversely, in old cultures, the increase in expression of the pro-inflammatory cytokine IL-1β suggests a shift towards the A1 activation type. A detailed characterization of A1 and A2 phenotypes of young and old astrocytes under a hypoxic state will help to expand our knowledge about adaptive capacity of the CNS and can be considered as an intriguing direction for upcoming research.

## 4. Materials and Methods

### 4.1. Research Object

The studies were performed in primary cultures of astrocytes obtained from the cerebral cortex of newborn C57BL/6 mice (P1-P6). Animals were housed in a certified SPF vivarium of the Lobachevsky State University of Nizhny Novgorod. The care and handling of the experimental animals adhered to the regulations outlined in the Rules of Laboratory Practice with the Use of Laboratory Animals (Russia, 2010) and the International Guiding Principles (Code of Ethics) for Biomedical Research Involving Animals (CIOMS and ICLAS, 2012). Moreover, the ethical principles established by the European Convention for the Protection of Vertebrate Animals used for Experimental and Other Scientific Purposes (Strasbourg, 2006) were also respected. Experimental procedures were approved by the Bioethics Committee of Lobachevsky University (ethics code No. 61 from 24 January 2022).

### 4.2. Isolation and Cultivation of Primary Cultures of Astrocytes

Primary cultures of cortical astrocytes were obtained following the protocol described in [10]. Briefly, the surgically isolated cerebral cortex of newborn mice (P1-P6) was cleared of the meninges and then subjected to mechanical and enzymatic dissociation using a 0.25% trypsin-EDTA solution (Thermo Fisher Scientific, Waltham, MA, USA). Following 20-min dissociation in a CO_2_ incubator (Binder C150, BINDER GmbH, Tuttlingen, Germany), the cell suspension was washed three times with a Ca^2+^- and Mg^2+^-free phosphate-buffered saline (PBS, Thermo Fisher Scientific, Waltham, MA, USA) and then centrifuged in a culture medium (DMEM (Dulbecco’s Modified Eagle Medium), Thermo Fisher, Waltham, MA, USA) supplemented with 10% fetal bovine serum (PanEco, Moscow, Russia), 1% sodium pyruvate (Thermo Fisher, Waltham, MA, USA), 1% B27 supplement (Thermo Fisher Scientific, Waltham, MA, USA), and 0.5% L-glutamine (Thermo Fisher Scientific, Waltham, MA, USA). The cell suspension was then centrifuged at 1000 rpm for 3 min, and the resulting pellet was seeded onto 24-well culture plates previously coated with a polyethyleneimine solution (1 μg/mL) (Merck KGaA, Darmstadt, Germany) to promote efficient cell attachment to the substrate. The cultures were passaged on day 7 after the start of cultivation. The cells were removed from the substate with a trypsin–versine solution (1:3) (PanEco, Moscow, Russia), and after the subsequent washing step and centrifugation they were then reseeded at an approximate density of 4500 cells per mm^2^. Following this protocol, the elimination of neuronal population from the cultures is achieved, as shown in our previous study [10].

The control astrocyte cultures composing the “Intact astrocytes” group (n = 33) were cultivated for 21 days in vitro (DIV) in a CO_2_ incubator, while the viability of cultures subjected to physiological aging were maintained for 157 DIV (n = 17). The morphological state of the primary astrocyte cultures was monitored on days of culture medium replacement using an Axio Observer A1 (Zeiss, Oberkochen, Germany) inverted microscope.

### 4.3. In Vitro Hypoxia Model

The simulation of acute normobaric hypoxia was proceeded on DIV 14 for the “young” group of cultures (“Hypoxia young”, n = 39), and on DIV 150 for the “old” group of cultures (“Hypoxia old”, n = 9). The procedure consists of replacing the conditioned culture medium with a medium containing a low oxygen concentration for 30 min, followed by the subsequent replacement with a complete growth medium. To obtain a hypoxic medium, argon gas at a pressure of 1–1.5 MPa was passed through the culture medium with normal oxygen content in a sealed chamber [10,57].

### 4.4. Calcium Imaging

The imaging studies of functional calcium activity in primary cortical astrocytes, demonstrating the functional state of calcium homeostasis in cells, were performed using a Zeiss LSM 800 confocal laser scanning microscope (Carl Zeiss, Oberkochen, Germany). The Oregon Green 488 BAPTA-1 AM (OGB-1, 0.4 μM, Invitrogen, Thermo Fisher Scientific, Waltham, MA, USA) calcium sensor was dissolved in dimethyl sulfoxide (Sigma-Aldrich, Merck KGaA, Darmstadt, Germany) supplemented with 4% pluronic F-127 (Invitrogen, Thermo Fisher Scientific, Waltham, MA, USA), followed by addition to the culture medium and incubation under conditions of a CO_2_ incubator for 30 min. The stained cultures were then examined by confocal microscopy. For excitation, a highly efficient LED with λ = 488 nm was used. Fluorescence emission was recorded using a light filter with a bandwidth of 500–550 nm. A time series of images were acquired to capture the dynamics of intracellular calcium concentration changes. The recording frequency was set at 2 frames per second. The calcium activity of primary astrocyte cultures was recorded on day 7 after hypoxia modeling, i.e., at 21 DIV for the “young” astrocyte group and 157 DIV for the “old” astrocyte group, respectively [10,11]. This approach allowed the acquisition of 99 video streams, each with a field of view size of 639 × 639 µm (or frame size of 512 × 512 pixels), and a duration of 20 min.

### 4.5. Building a Dynamic Graph and Definition of Network Connectivity Metrics

To study intercellular Ca^2+^ signaling during aging and hypoxia modeling, the network characteristics of calcium dynamics were assessed using an original algorithm for analyzing data on the calcium activity of cell cultures. This algorithm, initially developed to detect calcium events, was implemented in the Matlab R2021b (The Math Works, Inc.) [58]. It was further adapted and integrated into the AstroLab original software program (computer program registration certificate No. 2021612870, dated 25 February 2021), as described in detail in [10].

Network analysis is a powerful tool to provide deeper insights into the Ca^2+^ propagation between cells. The transmissions between cells in one particular moment of time can be represented as a directed graph with nodes corresponding to cells and directed links—to signal propagation from one cell to another. Applying this idea to the whole video of neuro-glial calcium activity, we obtain a series of graphs called dynamic graphs.

The main step of the suggested algorithm is the identification of calcium transmissions between cells. Due to low spatial precision in the case of hundreds of cells in the field of view, obtaining exact transmissions is challenging. To overcome this issue, we defined the transmission as co-occurring events with a link directed from the cell with an earlier event start to the cell with a later event start. Co-occurring means the events have joint action time. Finally, the event starts and ends were a point in time where calcium intensity exceeds their slow exponential moving average (EMA) line computed in the forward direction for starts and backward for ends. In addition, events are defined as local peaks that exceed the exponential moving average of the calcium intensity fluctuations.

Based on the dynamic graph we introduced the compressed graph over time, which results in a weighted directed graph that characterizes the number of registered transmissions between each pair of cells. The obtained graph represents the overall activity in a network and highlights the most frequent paths of transmissions between cells.

According to network theory, two groups can be distinguished among cells: hubs and authorities. A part of cells can mostly integrate information from many other cells and some cells can mostly transmit information to other cells. Cell-integrators are called authorities and cell-transmitters are called hubs. However, cells can be important only in case if they send or receive information from other important cells. In the network theory, the quantity of “hubness” and “authority” of cells is introduced by some real values between 0 and 1. Higher values of “hubness” are assigned to cells that are transmitted to many cells that have high values of “authority” (i.e., cell integrators). Higher values of “authority” of a cell mean that many cells with high “hubness” transmit to it.

Based on the generated dynamic and compressed graphs, we determined the following metrics [11]:

- The number of edges in a dynamic graph. This metric represents the total count of edges observed across all frames. It takes into account the presence of edges in each frame, considering the entire overlapping time of the compared events:

(1) $$\left|E_{D}\right|=\sum_{t=1}^{T}\left|E_{t}\right|$$

where $E_{D}$ is the set of dynamic graph edges, $E_{t}$ is the set of t-frame graph edges, T–the number of frames.

- The average number of periodically occurring connections per cell. This metric specifically considers the links that occur more than once throughout the entire video:

(2) $$\frac{\sum_{v_{i}\in V_{D}}^{}\left(\deg^{-}(v_{i})+\deg^{+}(v_{i})\right)}{n}$$

where $V_{D}$ is the set of dynamic graph vertices, $v_{i}$—*i* vertex, *n*—number of vertices.

- Hubness is a measure of a cell’s mediation score, which is computed as the sum of the authority scores of the cells that follow it in the transmission chain. Mediation rating can be gained by transmitting signals to multiple cells, or by transmitting signals to one significant cell:

(3) $$hub(v)=\sum_{v\in V_{from}}auth(v)$$

where $V_{from}$ is the set of vertices from which the signal propagated to vertex v.

- Authority represents a cell’s significance score, determined by summing the hubness scores of cells preceding it in the transmission chain. A cell can obtain a higher authority score by receiving signals from multiple cells or accepting signals from a highly rated hub:

(4) $$auth(v)=\sum_{v\in V_{to}}hub(v)$$

where $V_{to}$ is the set of vertices to which the signal propagated from vertex v.

We employed the method of constructing group histograms to analyze the activity of cells from multiple cultures within the same group. Since each culture within a group may exhibit different activity patterns, the group histogram serves as a valuable tool for summarizing the activity characteristics of cells across different cultures, thereby creating a collective representation or “group portrait” of the activity [11].

### 4.6. Quantitative Real-Time PCR (RT-qPCR)

Total RNA from primary cortical astrocytes was isolated using the ExtractRNA reagent (Eurogen, Moscow, Russia) following the manufacturer’s instructions. The amount of isolated RNA was determined using UV spectrophotometry (Nanodrop One, ThermoFisher Scientific, Waltham, MA, USA). For cDNA synthesis, the MMLV RT kit and random primer (Eurogen, Moscow, Russia) were used.

The following pairs of primers were used for the real-time PCR:

Oaz1_fw 5′-AAGGACAGTTTTGCAGCTCTCC-3′,

Oaz1_rv 5′-TCTGTCCTCACGGTTCTTGGG-3′;

Bcl-2_fw 5′-CTACGAGTGGGATGCTGGAGATG-3′,

Bcl-2_ rv 5′-TCAGGCTGGAAGGAGAAGATGC-3′;

Gja1_fw 5′-TGCGCTTCTGGGTCCTTCAGAT-3′,

Gja1_rv 5′-CTGCGCCACTTTGAGCTCCTCT-3′;

Hif1α_fw 5′-GCAATTCTCCAAGCCCTCCAAG-3′,

Hif1α_rv 5′-TTCATCAGTGGTGGCAGTTGTG-3′;

Tnfa_fw 5′-GCCCACGTCGTAGCAAACC-3′

Tnfa_rv 5′-TGGTTGTCTTTGAGATCCATGCC-3′

Il1b_fw 5′-GCCCATCCTCTGTGACTCATGG-3′

Il1b_rv 5′-GTTCATCTCGGAGCCTGTAGTGC-3′

Il10_fw 5′-AAGCATGGCCCAGAAATCAAGG-3′

Il10_rv 5′-CAGGGGAGAAATCGATGACAGC-3′

Amplification protocol: 50 °C—2 min, 95 °C—10 min, cycling part (40 cycles) 95 °C—15 s, 60 °C—60 s on a QuantStudio 5 thermocycler (Applied Biosystems, ThermoFisher Scientific, Waltham, MA, USA) using reaction mixture qPCRmix-HS SYBR+LowROX (Eurogen, Moscow, Russia).

The data obtained were processed using the ΔΔCt method and a reference sample of cultures from the intact group that did not undergo hypoxia, in which the expression level was taken as a unit. The reference gene (Oaz1) was used for data normalization.

### 4.7. Statistical Analysis

The data obtained were analyzed for normal distribution using the Shapiro–Wilk test. If the data followed a normal distribution, the results are presented as mean ± standard error of the mean (m ± SEM). On the other hand, if the data did not follow a normal distribution, they are presented as “M [Q1; Q3]”, where M represents the median, Q1 is the first quartile (quantile 0.25), and Q3 is the third quartile (quantile 0.75) of the samples in the group. For normally distributed data, the statistical significance of differences between samples was determined using the one-way ANOVA test. The nonparametric Mann–Whitney test or Kruskal–Wallis test for multiple comparisons was used to assess the significance of differences between groups with a distribution other than normal. Differences between groups were considered significant at *p* ≤ 0.05.

## 5. Conclusions

Novel algorithms were employed for the visualization and statistical analysis of various metrics related to the network calcium activity of astrocytes. These metrics included the number of signal transmissions between cells, the quantification of periodically formed functional connections, the assessment of mediation, and the significance of individual cells in signal transmission. Application of these algorithms allows for the identification of significant disruptions in intercellular signaling and, consequently, a decrease in the functional calcium activity of astrocytes during physiological aging and hypoxic damage in vitro.

## Data Availability

The data used to support the findings of this study are available from the corresponding author upon request.

## Supplementary Materials

The following supporting information can be downloaded at https://www.mdpi.com/article/10.3390/ijms241512286/s1.
