# Supplementary material for: Unravelling the Collective Calcium Dynamics of Physiologically Aged Astrocytes under a Hypoxic State In Vitro
Source: Int J Mol Sci. 2023 Jul 31;24(15):12286. doi: 10.3390/ijms241512286 (PMC10419080; doi:10.3390/ijms241512286)
Supplement: Supplementary file 1 [file ijms-24-12286-s001.zip › Supplementary materials.pdf]

Table S1. The number of signal transmissions between cells in primary cultures of cortical astrocytes.

Number of signal transmissions / Group	Intact young	Hypoxia young	Intact old	Hypoxia old
From 1 to 20	0.00 [0.00; 0.12]	0.00 [0.00; 0.76]	3.20 [1.23; 6.60]	6.58 [3.62; 8.71]
From 21 to 44	0.00 [0.00; 0.37]	0.00 [0.00; 1.44]	1.01 [0.00; 5.26]	0.00 [0.00; 0.91]
From 45 to 150	0.00 [0.00; 7.39]	1.56 [0.00; 16.34]	0.00 [0.00; 3.58]	0.00 [0.00; 0.00]
From 151 to 400	1.84 [0.65; 4.41]	3.06 [0.64; 16.43]	0.00 [0.00; 0.00]	0.00 [0.00; 0.00]
From 401 to 7,500	27.26 [2.59; 55.66]	44.24 [2.65; 74.48]	0.00 [0.00; 0.00]	0.00 [0.00; 0.00]
From 7,501 to 30,000	31.96 [0.00; 54.86]	0.00 [0.0000; 4.81]	0.00 [0.00; 0.00]	0.00 [0.00; 0.00]

Data are presented as "M [Q1; Q3]", where M represents the median, Q1 is the first quartile (quantile 0.25), and Q3 is the third quartile (quantile 0.75) of the samples in the group
